# Hepatitis C Virus Infection among HIV-Infected Patients Attending Dessie Referral Hospital, Northeastern Ethiopia

**DOI:** 10.1155/2021/6675851

**Published:** 2021-01-22

**Authors:** Alemu Gedefie, Aderaw Adamu, Ermiyas Alemayehu, Yeshimebet Kassa, Melaku Ashagrie Belete

**Affiliations:** Department of Medical Laboratory Science, College of Medicine and Health Sciences, Wollo University, Dessie, Ethiopia

## Abstract

**Objective:**

Hepatitis C Virus (HCV) and Human Immunodeficiency Virus (HIV) coinfection increases the incidence of end-stage liver disease which is more severe in immune-compromised HIV-infected patients than HCV infection alone. The aim of this study was to assess HCV infection and the associated risk factors among HIV/AIDS patients attending Dessie Referral Hospital, Northeastern Ethiopia.

**Methods:**

A hospital-based cross-sectional study was conducted among 249 HIV-infected adults selected by a systematic random sampling technique from January to March 2018. A structured questionnaire was used to collect sociodemographic and risk factor data. Moreover, the blood specimen was collected and tested for CD4 count and anti-HCV antibody detection according to standard operating procedures. The data obtained were entered into SPSS version 20, and descriptive statistics, bivariate and multivariate logistic regression analyses were performed. A *P* value ≤0.05 with a corresponding 95% confidence interval was considered as statistically significant.

**Result:**

Of a total of 249 HIV-infected study subjects, 120 (48.2%) were male and 129 (51.8%) were females, while the mean (±SD) age and CD4+ cells/mm3 were 39.10 (±11.507) years and 316.08 + 290.607 cells/mm3, respectively. Anti-HCV antibody was detected in 13 (5.2%) patients with higher prevalence rate found in males (*P*=0.078) and elders >50 years of age (*P*=0.013) than their counterparts. Age group of >50 years of age (AOR = 9.070, 95% CI: 1.578, 52.117, *P*=0.013), longer duration of HIV treatment (AOR = 5.490, 95% CI: 1.341, 34.458, *P*=0.041), WHO clinical stage III/IV (AOR = 12.768, 95% CI: 2.293, 71.106, *P*=0.004), previous history of hospitalization (AOR = 10.234, 95% CI: 2.049, 51.118, *P*=0.005), tooth extraction (AOR = 6.016, 95% CI: 1.137, 36.837, *P*=0.048), and liver disease (AOR = 11.398, 95% CI: 1.275, 101.930, *P*=0.029) were statistically significant predictors of HCV infection.

**Conclusion:**

The prevalence of HCV infection is still higher and causes concern. Therefore, screening of these high-risk groups should be critical to reduce mortality and to improve clinical outcomes.

## 1. Background

Hepatitis C Virus (HCV) was first recognized in 1989 to be a cause of acute and chronic hepatitis related to transfusion [1]. HCV is the second major viral infection next to human immunodeficiency virus (HIV) for more than two decades [2] which becomes a major public health problem causing estimated annual deaths of 350,000 patients worldwide [1]. Globally, an estimated 71 million people are chronically infected with HCV globally [3], of which 5 million people are coinfected with both HIV and HCV [4]. The prevalence of HIV/HCV coinfection (HCV among HIV-infected patients) is higher in developing nations which disproportionally varies geographically in sub-Saharan Africa from 0% to 22% [5].

HCV is associated with chronic liver disease leading to cirrhosis as well as hepatocellular carcinoma; about 20% of patients with chronic HCV infection are more prone to develop cirrhosis over an interval of 20 to 50 years [6]. HIV/HCV coinfection is known to increase the incidence of end-stage liver disease which is more severe in immunosuppressed patients infected with HIV than those who have HCV infection alone [7, 8]; as a result, HIV-infected individuals are at risk of being coinfected with HCV. HIV suppresses the immune system of an infected host which creates a conducive environment for HCV to lower viral clearance of HCV infection, increase levels of HCV RNA in body fluid, aggravate progression of infection to HCV-related end-stage liver disease, and increase risk and frequency of hepatotoxicity of antiretroviral treatment (ART) [9, 10].

Appropriate management and monitoring are needed to improve the quality of treatment and increase life expectancy of HIV-infected patients coinfected with HCV [11]. Hence, the magnitude and predisposing risk factors should be well investigated to design strategies and take measures for reduction of such comorbidities. Besides, all HIV patients should be routinely tested for markers of HCV infection, and emphasis should be given towards the advantage of early detection and therapy to reduce morbidity and mortality rate. Despite these facts, little attention is given about HCV/HIV coinfections in Ethiopia, and the recent ART guidelines of the country recommends only a liver function biochemical test, the alanine amino transferase (ALT) level test, to identify liver-related complications among HIV patients rather than routine screening tests for HCV [12].

Certain studies have been conducted in Ethiopia, but the magnitude of the problem and the predisposing factors are not well addressed and little is known about HCV/HIV coinfection [13] including the rate of coinfection in our setting, Northeastern Ethiopia. Therefore, the aim of this study was to assess the seroprevalence of HCV infections and associated risk factors among HIV-infected patients on ART at Dessie Referral Hospital, Northeastern Ethiopia.

## 2. Method and Materials

### 2.1. Study Design and Setting

This hospital-based cross-sectional study was conducted from January to March 2018 among HIV-infected adults attending the ART follow-up clinic of Dessie Referral Hospital, Northeastern Ethiopia. Dessie town is located 401 Km Northeast of Addis Ababa, the capital of Ethiopia. According to the study by the central statistical agency of Ethiopia conducted in 2007, the population of Dessie town was estimated to be about 151,174, of which about 120, 095 or 79.44% are urban inhabitants living in the town of Dessie. Dessie Referral Hospital is serving as a referral center for the population of Northeastern Amhara and Afar region and has a bed capacity of 300. The hospital had a total of 5941 registered HIV-infected patients on follow-up at the ART follow-up clinic.

### 2.2. Sample Size Determination and Sampling Technique

All selected HIV-infected adults who were registered and currently attending the ART clinic of the hospital for follow-up during the study period were included in this study after the minimum sample size was determined by using a single population proportion formula considering the prevalence of HCV among HIV patients 10.5% [14], marginal error of 4%, and 95% confidence interval = 1.96 by using the following sample size determination formula:

(1) $$\begin{array}{c} n=\frac{Z^{2}XP\left(1-P\right)}{D}, \end{array}$$

where *n* = the minimum sample size required, *Z*_*a*/2_ = the significant value for 95% confidence interval, *P* = the expected coinfection of HCV and HIV patients [14], and *d* = the margin of error.

By including 10% nonresponse rate, 249 study subjects were selected by the systematic random sampling technique.

### 2.3. Sociodemographic and Laboratory Data Collection and the Analysis Procedure

All necessary information was collected from HIV-infected individuals on ART who fulfill the inclusion criteria using a structured questionnaire intended for collecting sociodemographic characteristics and other potential risk factor data after written informed consent was obtained.

8 ml of venous blood, 4 ml with EDTA anticoagulant and 4 ml in plain (without anticoagulant) test tubes containing gel, was collected through standard venipuncture techniques from study participants on the spot strictly following the standard operating procedure. A portion of the EDTA anticoagulated blood was used for automated analysis of CD4/CD3 cells/mm^3^. About 4 ml of nonanticoagulated blood was left without any disturbance for about 30 to 60 min for complete clot formation, and then, serum was extracted by centrifugation at 3000 rpm for 10 min. A clear nonhemolyzed extracted serum was analyzed for anti-HCV antibody detection using anti-HCV rapid test kits (Zhejiang Orient Gene Biotech Co., LTD., China) according to the manufacturer's instructions.

### 2.4. Anti-HCV Antibody Detection and CD4 Determination

An HCV test was performed for the presence of anti-HCV using rapid test strips (Zhejiang Orient Gene Biotech Co., LTD, China) that uses serum and/or plasma and utilizes the lateral flow chromatographic immunoassay-based principle of the double antigen-sandwich technique. An automated analysis of CD4/CD3 cells/mm^3^ was carried out using the BD-FACS count machine (Becton Dickinson, San Jose, CA, USA).

### 2.5. Quality Assurance

The quality of laboratory test results was assured by strictly following standard operational procedures during laboratory investigation, and the requirements related to proper use of all equipment, reagents, and controls were followed according to the standards on the manufacturer's instructions. The analysis (control) was run together with patient samples without giving any special considerations using known anti-HCV antibody-positive and anti-HCV antibody-negative samples. Finally, appropriate test procedures and interpretation of test results were performed according to the precautions and instructions supplied by the manufacturer.

### 2.6. Data Analysis Procedure

All data in the study were checked for completeness and entered into EPI data followed by transferring of data into Statistical Packages for Social Science (SPSS) version 20 software (IBM, USA) for analysis. The data were organized and summarized using descriptive statistics including frequencies, percentages, and mean as needed. Binary logistic regression was used to show the association of each variable with the dependent variable. Moreover, a multivariate analysis was computed to identify factors that independently influence the occurrence of the dependent variable. A *P* value <0.05 with 95% confidence interval was considered statistically significant.

### 2.7. Ethical Considerations

Ethical clearance was obtained from the ethical review committee of Wollo University. Permission was obtained from the Dessie Referral Hospital research committee through a formal letter written from Wollo University. Informed consent was obtained from each study participants. Study subjects who have abnormal finding were referred to the attending physician for appropriate treatment.

## 3. Result

A total 249 HIV-infected study participants were included in this study with a response rate of 100%. The mean (±SD) age of the study participants was 39.10 (±11.507) years with a proportion of 120 (48.2%) male and 129 (51.8%) female participants. Of the total participants, 166 (66.7%) were rural dwellers, 157 (63.1%) were married, 73 (29.3%) were illiterate, and nearly half, 45%, of the participants had an average monthly income level of less than <13 US dollars (Table 1).

With regard to duration of HIV infection from diagnosis and duration of HIV treatment, about 173 (69.5%) and 69 (27.7%) HIV patients had more than 10 years of duration, respectively. The mean (±SD) of CD4 cells/mm^3^ was 316.08 ± 290.607. Among all participants, 112 (45%) were on WHO clinical stage II, 111 (44.6%) had CD4 count of less than 200 cells/mm^3^, and 12 (4.8%) had a history of liver disease (Table 1).

Of the total 249 HIV-infected study participants, anti-HCV antibody was detected in 13 (5.2%) patients. Despite being statistically insignificant, there was higher prevalence of HCV, 7 (9.6%) among illiterate participants and 12 (6.9%) among patients with more than 10 years of duration of HIV infection, in which *P* value >0.05 in both cases. In such instance, the odds of study participants with more than 10 years of duration of HIV infection (AOR = 4.934, 95% CI: 0.463, 52.548, *P*=0.186) was around five times higher for acquiring HCV infection than its counterpart. On the contrary, there was significantly higher prevalence of HCV among study participants in the age group of >50 years of age (AOR = 9.070, 95% CI: 1.578, 52.117, *P*=0.013) than participants aged below 50 years. Likewise, participants with more than 10 years of duration of HIV treatment (AOR = 5.490, 95% CI: 1.341, 34.458, *P*=0.041) and those who are on WHO clinical stage III/IV (AOR = 12.768, 95% CI: 2.293, 71.106, *P*=0.004) were more likely to acquire HCV infection (Table 2). The prevalence of HCV among HIV-infected patients in this study was significantly higher among participants who had a previous history of hospital admission (AOR = 10.234, 95% CI: 2.049, 51.118, *P*=0.005), tooth extraction (AOR = 6.016, 95% CI: 1.137, 36.837, *P*=0.048), and liver disease (AOR = 11.398, 95% CI: 1.275, 101.930, *P*=0.029) (Table 2).

## 4. Discussion

In peoples living with HIV, liver disease is an important modifier of health [15]; and HCV infections are a major contributor for an increased morbidity and mortality rate among HIV patients that lead to rapid progression to AIDS, hepatocellular carcinoma [16], and end-stage liver disease [17]. Therefore, investigation of HCV infection in HIV-infected patients is very important to prevent them from further infections and complications [18]. In line with this, the present study showed an overall prevalence (5.2%) of HCV among HIV-infected adults who were on ART, which indicates that HCV infection is still the major public health problem in Ethiopia.

The finding of the present study (5.2%) was in agreement with HIV-HCV coinfection rates of HIV-positive individuals documented in previous studies of Northwestern Ethiopia, 5.0% in Gondar [19] and 5.4% in Burkina Faso [20]. However, the HCV/HIV coinfection rate was higher than the findings of studies conducted in Cameroon, 2.8%, [21] and Debretabor Hospital, Ethiopia, 1.3%, [13]. The presence of shared modes of transmission of both viruses in the study patients [22, 23] as well as limited access of health information about the transmission and prevention of HCV infection in the study area might be possible reasons for the discrepancies seen in this study.

The finding of this study was relatively lower than that of the studies conducted in Nigeria, 8.2%, [24] and Ethiopian studies such as those in Bahir Dar, 18.9%, [25], Adewa, 6.6%, [26], Hawassa, 10.5%, [14] Mekelle, 8.6%, [27], and Addis Ababa, 11.6%, [22] which might be due to differences in geographic regions, types of risk exposure, methodology used [28, 29], time period variation, and reduced proportion of high-risk groups such as intravenous drug users in the study area. Additionally, this study did not use Enzyme-Linked Immuno-Sorbent Assay (ELISA) and Quantitative-real time Polymerase Chain Reaction (qRT-PCR) which might underestimate the detection rate of the viral infection in this study.

The prevalence rate of HCV in this study was significantly higher among participants aged >50 years (*P*=0.013), which is different from a study report in Guinea-Bissau [30]. This might be due to end-stage liver disease complications [31]. Moreover, in this study, the prevalence of HCV infection among HIV-infected patients who were on ART for more than 10 years of duration was significantly higher (*P*=0.041). This is mainly attributed to a higher incidence of adverse drug reactions; hepatotoxicity due to ART drugs as their stay on ART increased [32].

HIV patients who were on WHO clinical stage III and/or IV were more likely to develop HCV infection which agreed with previous reports of a study in Vietnam that showed a statistically significant association of advanced clinical stage classification with HCV infection [33]. On the contrary, the present finding disagreed with a study conducted in Cameroon [21]. The present study also showed that participants who had a pervious history of liver disease are at increased risk of developing HCV infection than their counter parts, which contradicted with a study conducted in Cameroon [21] and Southern Ethiopia, Hawassa [14]. On the other hand, this study showed a statistically significant association of previous history of hospitalization and tooth extraction with HCV infection among HIV patients, which is in agreement with reports from Ethiopia: Gondar [13] and Mekelle [27].

Due to lack of resources in this research; ELISA and PCR tests were not used that can detect early HCV infections than the Rapid test kit. Therefore, the finding of the present study might be underestimated.

## 5. Conclusions

The prevalence of HCV infection is lower than most of the previous reports from Ethiopian studies. Longer duration of HIV treatment, advanced WHO clinical stage, previous history of hospitalization, tooth extraction, and history of liver disease were significantly associated with HCV infection. Thus, routine diagnosis of HCV among HIV patients should be advocated to be included as an essential component of the ART-monitoring strategy for improved patient care. Moreover, effective management of viral hepatitis should now be considered a priority in HIV/HCV coinfected patients to reduce mortality and to improve clinical outcomes.

## Figures and Tables

**Table 1:** Sociodemographic and clinical characteristic of HIV/AIDS patients attending ART  clinic of Dessie Referral Hospital, Northeastern Ethiopia, 2018 (*N* = 249).

Characteristics		Frequency	Percent
Age	18–29 years	42	16.9
	30–39 years	95	38.2
	40–49 years	64	25.7
	≥50 years	48	19.3

Sex	Male	119	47.8
	Female	130	52.2

Residence	Rural	166	66.7
	Urban	83	33.3

Marital status	Married	3	1.2
	Divorced	45	18.1
	Widowed	157	63.1
	Single	44	17.7

Educational status	Illiterate	73	29.3
	Primary	95	38.2
	Secondary	55	22.1
	Tertiary	26	10.4

Occupation	Merchant	43	17.3
	Student	70	28.1
	Housewife	47	18.9
	Daily labor	27	10.8
	Farmer	32	12.9
	Gov't employee	30	12.0

Monthly family income	<13 USD	112	45.0
	>13 USD	137	55.0

Duration of HIV infection from diagnosis	<5 years	14	5.6
	5–10 years	62	24.9
	>10 years	173	69.5

Duration of HIV treatment	<5 years	69	27.7
	5–10 years	111	44.6
	>10 years	69	27.7

WHO clinical staging	Stage 1	77	30.9
	Stage 2	112	45.0
	Stage 3	42	16.9
	Stage 4	18	7.2

Liver disease	Yes	12	4.8
	No	237	95.2

CD4^+^ category, cells/mm^3^	<200	111	44.6
	200–350	45	18.1
	>350	93	37.3

CD4^+^: cluster of differentiation 4; USD: United States Dollar; WHO: World Health Organization.

**Table 2:** Anti-HCV positivity in relation to HCV risk factors and sociodemographic factors among HIV-infected patients at Dessie Referral Hospital, Northeastern Ethiopia, 2018.

Variables	HCV antibody		COR (95% CI)	*P* value	AOR (95%CI)	*P* value
	Positive no. (%)	Negative no. (%)				
*Knowledge of HCV causative agent*						
Yes	3(3.2)	90(96.8)	1			
No	10(6.4)	146(93.6)	2.055 (0.551, 7.666)	0.284	NA	

*Knowledge of the mode of transmission*						
Yes	1(2.0)	49(98.0)	1			
No	12(6.0)	187(94.0)	3.144 (0.399, 24.772)	0.277	NA	

*History of hospitalization*						
Yes	10(13.2)	66(86.8)	8.586 (2.291, 32.177)	0.001	10.234 (2.049, 51.118)	0.005^*∗*^
No	3(1.7)	170(98.3)	1			

*Previous surgical history*						
Yes	2(8.3)	22(91.7)	1.769 (0.368, 8.495)	0.476	NA	
No	11(4.9)	214(95.1)	1			

*History of skin disorder*						
Yes	5(5.6)	85(94.4)	1.110 (0.352, 3.501)	0.858	NA	
No	8(5.0)	151(95.0)	1			

*History of circumcision*						
Yes	7(5.1)	131(94.9)	0.935 (0.305, 2.867)	0.907	NA	
No	6(5.4)	105(94.6)	1			

*History of blood transfusion*						
Yes	3(5.3)	54(94.7)	1.011 (0.269, 3.806)	0.987	NA	
No	10(5.2)	182(94.8)	1			

*History of unsafe sexual intercourse*						
Yes	6(6.3)	89(93.7)	1.416 (0.461, 4.347)	0.544	NA	
No	7(4.5)	147(95.5)	1			

*History of tooth extraction*						
Yes	4(17.4)	19(82.6)	5.076 (1.429, 18.034)	0.012	6.016 (1.137, 36.837)	0.048^*∗*^
No	9(4.0)	217(96.0)	1			

*Sharing of sharp material with friends*						
Yes	1(7.1)	13(92.9)	1.429 (0.172, 11.853)	0.741	NA	
No	12(5.1)	223(94.9)	1			

*Smoking habit*						
Yes	2(8.3)	22(91.7)	1.606 (0.336, 7.679)	0.553	NA	
No	13(5.8)	212(94.2)	1			

*Drinking habit*						
Yes	3(4.9)	58(95.1)	0.921 (0.245, 3.460)	0.903	NA	
No	10(5.3)	178(94.7)	1			

*Liver disease*						
Yes	3(25)	9(75)	7.567 (1.771, 32.323)	0.006	11.398 (1.275, 101.930)	0.029^*∗*^
No	10(4.2)	227(95.8)	1			

*Duration of HIV infection from diagnosis*						
>10 years	12(6.9)	161(93.1)	5.590 (0.714, 43.786)	0.101	4.934 (0.463, 52.548)	0.186
<10 years	1(1.3)	75(98.7)	1			

*Duration of HIV treatment*						
>10 years	8(11.6)	61(88.4)	3.905 (1.241, 34.546)	0.021	5.490 (1.341, 34.458)	0.041^*∗*^
<10 years	5(2.8)	175(97.2)	1			

*WHO clinical stage*						
I/II	5(2.6)	184(97.4)	1			
III/IV	8(13.3)	52(86.7)	5.662 (1.777, 18.041)	0.003	12.768 (2.293, 71.106)	0.004^*∗*^

*CD4+ category, cells/mm* ^*3*^						
≤200	5(4.5)	106(95.5)	0.767 (0.244, 2.412)	0.649	0.559 (0.120, 2.598)	0.458
>200	8(5.8)	130(94.2)	1			

*Sex*						
Male	10(8.4)	109(91.6)	3.884 (1.042, 14.471)	0.043	4.555 (0.842, 24.655)	0.078
Female	3(2.3)	127(97.7)	1			

*Age*						
≤50 years	9(4.4)	208(95.9)	1			
>50 years	4(12.5)	28(87.5)	3.302 (0.953, 11.433)	0.059	9.070 (1.578, 52.117)	0.013^*∗*^

^*∗*^Statistically significant at *P* ≤ 0.05. AOR: adjusted odds ratio, COR: crude odds ratio, 1: reference group, 95% CI: 95% confidence interval, HCV: Hepatitis C Virus, HIV: Human Immunodeficiency Virus, NA: not applicable, WHO: World Health organization.

## Data Availability

The data can be obtained from the corresponding author.

## Abbreviations

AOR: Adjusted odds ratio
AIDS: Acquired Immune Deficiency Syndrome
ART: Antiretroviral therapy
CD4^+^: Cluster of differentiation
CI: Confidence interval
COR: Crude odds ratio
FACS: Fluorescence-activated cell sorting
HIV: Human Immunodeficiency Virus
HCV: Hepatitis C Virus
SOPs: Standard operating procedures
WHO: World Health Organization.
